# Rab5-independent activation and function of yeast Rab7-like protein, Ypt7p, in the AP-3 pathway

**DOI:** 10.1371/journal.pone.0210223

**Published:** 2019-01-25

**Authors:** Hiroki Shimamura, Makoto Nagano, Keita Nakajima, Junko Y. Toshima, Jiro Toshima

**Affiliations:** 1 Department of Biological Science and Technology, Tokyo University of Science, Niijyuku, Katsushika-ku, Tokyo, Japan; 2 School of Health Science, Tokyo University of Technology, Nishikamada, Ota-ku, Tokyo, Japan; Iowa State University, UNITED STATES

## Abstract

The small GTPases, Rab5 and Rab7, are key regulators at multiple stages of the endocytic/endolysosomal pathway, including fusion and maturation of endosomes. In yeast, Vps21p (Rab5 homolog) recruits a GEF for Rab7 and activates the downstream Ypt7p (Rab7 homolog) on endosomal membrane. Although the model of this sequential activation from Vps21p to Ypt7p in the endocytic pathway has been established, activation mechanism of Ypt7p in the Vps21p-independent pathway has not been completely clarified. Here we show that Ypt7p is activated and mediates vacuolar fusion in cells lacking all yeast Rab5 genes, *VPS21*, *YPT52*, and *YPT53*. We also demonstrate that deletion of both *VPS21* and *YPT7* genes cause severe defect in the AP-3 pathway as well as the CPY pathway although the AP-3 pathway is mostly intact in each *vps21*Δ or *ypt7*Δ mutant. Interestingly, in *vps21*Δ *ypt7*Δ mutant cargos trafficked via the VPS or endocytic pathway accumulate beside nucleus whereas cargo trafficked via the AP-3 pathway disperse in the cytosol. These findings suggest that Ypt7p is activated and plays a Rab5-independent role in the AP-3-mediated pathway.

## Introduction

The Rab GTPases are important regulators of various intracellular vesicle transport systems. In the endocytic pathway, Rab5 has been shown to regulate the early steps involved in targeting endocytic vesicles to early endosomes and fusion between early endosomes [1–3]. Rab7 localizes to late endocytic compartments and plays important roles in transport from the early to late endosome and fusion of the endosome and lysosome/vacuole [4–7]. Recent studies have revealed that sequential activation or inactivation of Rab5 and Rab7, termed Rab conversion, along the endocytic pathway is important for achieving these roles [8–10]. In the Rab conversion process, Rab5 recruits Rab7-GEF to the endosome, thereby activating downstream Rab7, and this mechanism is well conserved in many organisms including budding yeast [11, 12].

In addition to these Rab proteins, two evolutionary conserved tethering complexes, the CORVET and HOPS complexes, are known to regulate early-to-late endosome transport, respectively [13]. These complexes share four class C subunits (Vps11p, Vps16p, Vps18p and Vps33p), and in addition to these subunits, CORVET contains Vps3p and Vps8p, and their homologous subunits Vps39 and Vps41 are contained in the HOPS complex [13]. Specific subunits of these tethering complexes interact with specific Rab protein: the Vps3p and Vps8p subunits bind to Vps21p at the endosomal membrane, whereas the Vps39p and Vps41p subunits bind to Ypt7p at the vacuolar membrane [14, 15]. The HOPS complex also interacts with the vacuolar SNARE complex (consisting of Vam3p, Vam7p, Vti1p and Nyv1p) via its class C subunits, and controls endosome-vacuole fusion [16]. Similarly, the CORVET complex is known to interact specifically with Pep12p, which functions as an endosomal t-SNARE required for transport from the TGN to the endosome [17], indicating that distinct sets of the tethering complex, SNARE, and Rab GTPase cooperatively mediate the early or late step of the endocytic pathway.

These proteins are also required for trafficking of newly synthesized proteins from the trans-Golgi network (TGN) to the vacuole via, for example, the vacuolar protein sorting (VPS) and adaptor protein (AP)-3 pathways [18–20]. Both of these pathways intersect with the endocytic pathway before transport to the vacuole [21], but the CPY pathway takes proteins via late endosomes/MVBs to the vacuole, whereas the AP-3 pathway mediates transport to the vacuole independently of late endosomes/MVBs. Several studies have identified around 80 mutants that have defects in the CPY pathway, and these *vps* mutants have been divided into six classes (A-F) based on their vacuolar morphology [22, 23]. Class D *vps* mutants, such as *vps21*Δ and *vps3*Δ, have a slightly enlarged vacuole, whereas class B *vps* mutants, such as *vps39*Δ, *vps41*Δ, have fragmented vacuole [22, 24]. Class C *vps* mutants, such as *vps33*Δ, contain only small vesicular remnants of a vacuole [22, 24], reflecting the roles of the class C Vps proteins in multiple stages of the vacuolar transport pathway [17]. Indeed, Vps33p is an essential core component of the CORVET and HOPS complexes, and a double mutant lacking endosomal SNARE Pep12p and vacuolar SNARE Vam3 exhibits a class C *vps* mutant phenotype [25].

Several Vps proteins, including Vps41p and Vam3, have been shown to function in the AP-3 pathway [26, 27]. Both Vps41p and Vam3p are required for homotypic vacuolar fusion [28, 29], but Vps41p seems to play more vital roles in the AP-3 pathway because it physically associates with an AP-3 subunit and mediates the formation of AP-3 transport vesicles [26]. In contrast to these proteins, Vps21p and Ypt7p seem to function mainly in the CPY pathway, because in *vps21*Δ and *ypt7*Δ mutants the AP-3 pathway is not severely impaired [21, 30, 31]. Although sequential activation of Vps21p and Ypt7p is reported to occur along the CPY pathway, *vps21*Δ and *ypt7*Δ mutants exhibit different vacuolar phenotypes, suggesting function of these proteins in the different vacuolar transport pathways and the existence of an unidentified mechanism that activates Ypt7p in the Vps21p-independent pathway.

In this study, we demonstrate that activated Ypt7p localizes to the vacuolar membrane in cells lacking all yeast Rab5 genes. We also show that the *vps21*Δ *ypt7*Δ double mutant exhibits a much severer vacuolar phenotype in comparison with the *vps21*Δ or *ypt7*Δ single mutant. Additionally, we show that the *vps21*Δ *ypt7*Δ mutant exhibits a severe defect in the AP-3 pathway. These results suggest that Ypt7p is activated and plays an important role in the Rab5-independent AP-3-mediated pathway.

## Materials and methods

### Yeast strains and growth conditions

The yeast strains used in this study are listed in S1 Table. All strains were grown in standard rich medium (YPD) or synthetic medium (SM) supplemented with 2% glucose and appropriate amino acids.

### Plasmids and strain construction

The N-terminal GFP tag was integrated at the endogenous locus of the *YPT7* gene as follows: The GFP (S65T) fragment whose stop codon was replaced with BglII site was subcloned into BamHI- and NotI-digested pBlueScript II SK (pBS-GFP), and the NotI-SacII fragment, which contains the *S*. *cerevisiae ADH1* terminator and the *His3MX6* module, was amplified by PCR using pFA6a-GFP (S65T)-HIS3MX6 as a template, and inserted into NotI- and SacII-digested pBS-GFP (pBS-GFP-HIS3). To create an integration plasmid, 395-bp 5' UTR of *YPT7* gene and the N-terminal fragment of the *YPT7* ORF (nt 1–288) were generated by PCR and cloned into the BamHI or BglII site of pBS-GFP-HIS3. To construct the plasmid expressing Ypt7p under the control of its own promoter (pRS316-*YPT7*), *YPT7* gene (containing 394 bp upstream and 172 bp downstream of the ORF) was amplified by PCR and cloned into the EcoRI-digested pRS316. To integrate GFP at the N terminus of the *YPT7* gene, the integration plasmid was linearized by HincII and transformed into yeast. The C-terminal GFP or mCherry tagging of proteins was performed as described previously [21].

### Fluorescence microscopy and electron microscopy

Fluorescence microscopy was performed using an Olympus IX83 microscope equipped with a x100/NA 1.40 (Olympus) objective and Orca-R2 cooled CCD camera (Hamamatsu), using Metamorph software (Universal Imaging). FM4-64 staining was performed as described previously [32]. The fluorescence intensities were analyzed by using the program ImageJ V1.44.

### Electron microscopy

Cells sandwiched between copper disks were frozen in liquid propane at -175°C and then freeze substituted with acetone containing 2% OsO_4_ and 2% distilled water at -80°C for 48 hr. The samples were kept at -20°C for 4 hr and then at 4°C for 1 hr, and dehydrated in anhydrous acetone two times and 100% ethanol three times. After being infiltrated with propylene oxide (PO) two times the samples were put into a 70:30 mixture of PO and resin (Quetol-651) and then transferred to a fresh 100% resin, and polymerized at 60°C for 48 hr. The blocks were cut into 70-nm-thick sections, and the sections were mounted on copper grids. The specimens were stained with 2% uranyl acetate and Lead stain solution, and observed using a transmission electron microscope (JEM-1400Plus; JEOL).

## Results

### *vps21*Δ *ypt7*Δ double mutant is a phenocopy of the class C *vps* mutant

**Fig 1:**
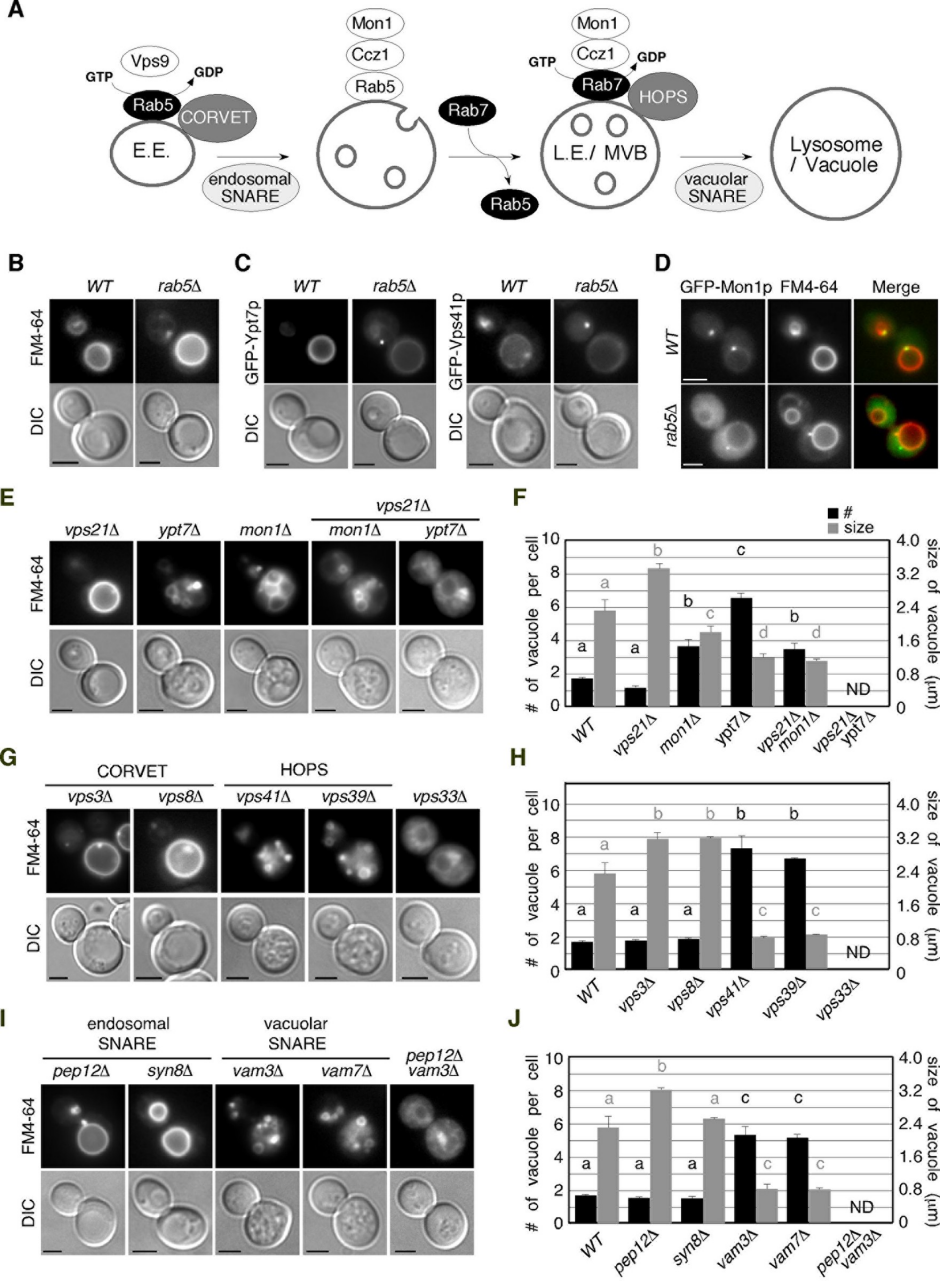
Vacuolar morphology of wild-type and mutant cells. (A) Model of Rab5 to Rab7 conversion during endosome maturation in budding yeast. Yeast Rab5 Vps21p is activated by Vps9p, a GEF for Rab5, on early endosome (E.E.). Activated Vps21p recruits Ccz1p/Mon1p, a GEF for Rab7, by biding GFP-bound Vps21p, and enhances dissociation of Vps21p form the early endosome. Ccz1p/Mon1p also binds to the HOPS complex and activates yeast Rab7 Ypt7p directly, thereby promoting endosome maturation and fusion of mutivesicular body/late endosome (MVB/L.E.) to the vacuole. Modified from Nordmann et al., 2010. (B, E, G, I) Vacuolar morphology in wild-type and indicated mutant cells. Vacuolar morphology was assessed by FM4-64 staining and different interference contrast (DIC) in the following strains: yeast Rab5-disrupted mutant (*rab5*Δ) (B), yeast Rab5 (*vps21*Δ), Rab7 (*ypt7*Δ) and Rab7-GEF mutants (*mon1*Δ)(E), CORVET and HOPS mutants (G), and SNARE mutants (I). (C) Locaization of GFP-Ypt7p, and -Vps41p in wild-type and *rab5*Δ cells. (D) Locaization of GFP-Mon1p in wild-type and *rab5*Δ cells. Cells were labeled with FM4-64. (F, H, J) Quantification of number (black bar) and size (gray bar) of vacuole in wild-type and mutant cells. Numbers were analyzed by counting ring-like compartments stained by FM4-64. Vacuolar size was measured inside diameter of the largest compartment stained by FM4-64 in cell. Data show the mean ± SD, with > 50 cells counted for each strain. Different letters indicate significant difference at *p* < 0.05 (S2 Table) (One-way ANOVA with Tukey’s post-hoc test). Scale bars, 2.5 μm.

**Fig 2:**
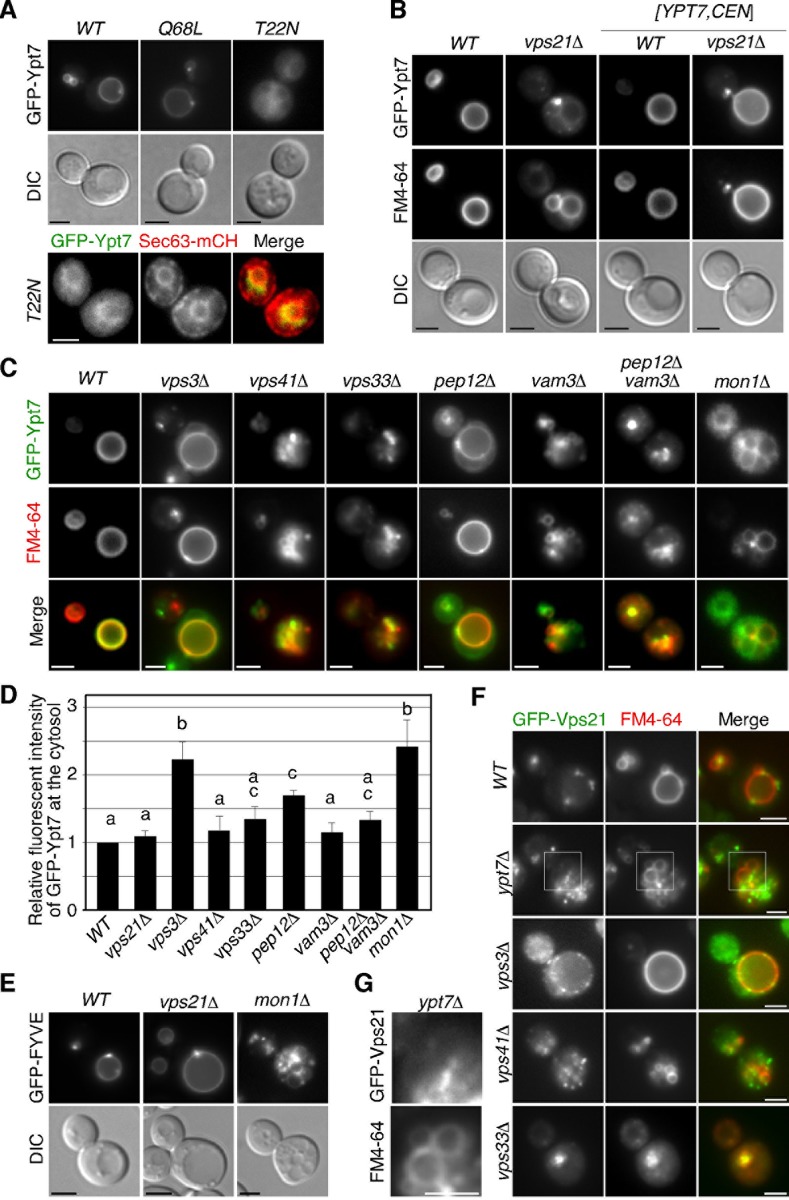
Localization of yeast Rab7 in wild-type and mutant cells. (A) Localization of putative GTP- or GDP-locked mutant of Ypy7p in living cells. Cells were grown to early to mid-logarithmic phase in YPD medium at 25°C and observed by fluorescence microscopy and DIC (upper panels). Colocalization of GFP-Ypt7(T22N) with mCherry-fused Sec63p (lower paneles). Each image pair was acquired at successive 2 sec (s) intervals. (B) Localization of GFP-Ypt7p in wild-type or *vps21*Δ cells. Cells were labeled with 200 μM FM4-64. Cells expressing Ypt7p from a single copy plasmid were grown to mid-logarithmic phase in selective synthetic rich medium at 25°C. (C) Localization of GFP-Ypt7p in wild-type and mutant cells. All cells express Ypt7p from a single copy plasmid. Cells were labeled with FM4-64 and observed as described above. (D) The graph shows quantification of the fluorescence intensity of GFP-Ypt7p in the cytosol. Error bars represent the SEM from at least three experiments (*n* > 50 cells for each strain per experiment). Different letters indicate significant difference at *p* < 0.05 (S2 Table) (One-way ANOVA with Tukey’s post-hoc test). (E) Localization of GFP-fused FYVE domain (EEA1) in wild-type and mutant cells. (F) Localization of GFP-Vps21p in wild-type and mutant cells. Cells were labeled with FM4-64. (G) Higher magnification views of the boxed areas in (F). Scale bars, 2.5 μm.

Vps21p has been reported to recruit the Mon1-Ccz1 complex, a GEF for Rab7, onto endosomes to activate Ypt7p during the early-to-late endosome transition (Fig 1A) [11]. According to this model, Vps21p is required for activation of Ypt7p and subsequent Ypt7p-mediated vacuolar fusion. Therefore it was speculated that cells lacking all of the yeast Rab5 genes, *VPS21*, *YPT52*, and *YPT53*, would show somewhat defect in the vacuole formation. However, a previous study reported that the *vps21*Δ *ypt52*Δ *ypt53*Δ mutant contained an enlarged vacuole, which is the typical morphology observed in the class D *vps* mutant, although the CPY pathway is severely impaired [31]. To confirm and further investigate these observations, we examined the vacuole morphologies of mutants harboring deletions of genes whose function is related to vacuole/endosome fusion by labeling the cells with a lipophilic styryl dye, FM4-64. When added to wild-type cells, FM4-64 is immediately incorporated into the plasma membrane, internalized via bulk-phase endocytosis, and then transported to the vacuole within 20 min (Fig 1B). As reported previously, we observed a slightly enlarged vacuole in the mother cell of the *vps21*Δ *ypt52*Δ *ypt53*Δ (*rab5*Δ) mutant (Fig 1B) [31]. To examine whether Ypt7p is recruited to the vacuolar membrane in the absence of yeast Rab5s, we determined the localization of Ypt7p using an N-terminal GFP-tagged protein expressed from the endogenous locus. Since GFP tagging partially perturbs the function of endogenous Ypt7p, we additionally expressed exogenous Ypt7p in cells expressing GFP-Ypt7p (see Fig 2B). GFP-Ypt7p was clearly detected on the vacuolar membrane in wild-type and the *rab5*Δ cells, although it showed partial relocation to the cytosol in the *rab5*Δ mutant (Fig 1C). We also found that Vps41p, a subunit of the HOPS complex, is able to stay on the vacuolar membrane (Fig 1C), whereas Mon1p, a GEF for Ypt7p, is mostly relocalized to the cytosol in the absence of yeast Rab5s, and prevacuolar localization observed in wild-type cell was significantly decreased (Fig 1D). These observations indicate that Ypt7p is able to partially localize on the vacuole, independently of yeast Rab5s. Quantitative analysis revealed that the number of vacuoles contained in wild-type cells averages ~1.7 and that the diameter of the largest vacuole is ~2.7 μm (Fig 1E and 1F). The *vps21*Δ mutant contains an enlarged vacuole (~3.3 μm) (Fig 1E and 1F), similar to the *rab5*Δ cells, whereas *ypt7*Δ cells exhibited severe vacuole fragmentation, the average size of the largest vacuole being ~1.2 μm (Fig 1E and 1F). Deletion of the *MON1* gene caused moderate fragmentation of the vacuole (~1.8 μm), and the *mon1*Δ and *vps21*Δ combination led to a slight decrease in size (~1.1 μm) (Fig 1E and 1F). Interestingly, we found that the *vps21*Δ *ypt7*Δ double mutant exhibits an apparently distinct vacuolar phenotype, in comparison with each single mutant; the cells lack a distinguishable vacuole and exhibit FM4-64 accumulation beside the nucleus (Fig 1E).

We also examined the morphology of the vacuole in mutants with deletion of the genes encoding the CORVET/HOPS or SNARE complex subunits. Deletion of the CORVET-specific Vps3p or Vps8p subunit resulted in a class D *vps* phenotype with an enlarged vacuole (~3.1 μm) similar to the *vps21*Δ mutant (Fig 1G and 1H) [24], whereas deletion of the HOPS-specific Vps39p or Vps41p subunit caused vacuolar fragmentation (~0.8 μm), characterized as the class B *vps* phenotype (Fig 1G and 1H) [24]. Cells with deletion of the *VPS33* gene, encoding a core subunit of two tethering complexes, exhibited severe defects in vacuolar morphology, categorized as the class C *vps* mutant (Fig 1G and 1H), consistent with previous reports [22]. Interestingly, we found that the *vps33*Δ mutant shows accumulation of FM4-64 beside the nucleus in addition to diffusion into the cytosol, similar to the *vps21*Δ *ypt7*Δ double mutant (Fig 1G).

Cells with deletion of *VAM3* or *VAM7*, encoding vacuolar t-SNARE, are categorized as the class B *vps* mutant and exhibit FM4-64 staining similar to the *vps39*Δ or *vps41*Δ mutant, whereas deletion of *PEP12* gene, encoding an endosomal t-SNARE, exhibited the class D *vps* phenotype (Fig 1I and 1J) [24]. Deletion of *SYN8* gene had little effect on the vacuolar morphology (Fig 1I and 1J). The *pep12*Δ *vam3*Δ double SNARE mutant exhibited a severe vacuolar phenotype comparable to that of the *vps33*Δ mutant [13]. Consistent with this, the *pep12*Δ *vam3*Δ mutant showed an FM4-64 staining pattern similar to the *vps33*Δ mutant (Fig 1I). These results demonstrated that the *vps21*Δ *ypt7*Δ mutant has a similar phenotype to the class C *vps* mutant.

### Membrane targeting of Ypt7p in *vps21*Δ or CORVET/HOPS complex mutants

We next focused on the active/inactive state of Ypt7p in mutant cells. It has been reported that substitution of a highly conserved asparagine by leucine (Q68L) or of a threonine by glutamine (T22N) fixes Rab proteins to the GTP- or GDP-bound form [33]. The GTP-bound active form of Ypt7p showed vacuolar membrane localization (Fig 2A, upper center panels). In contrast, the GDP-bound form of Ypt7p, Ypt7(T22N)p, exhibited localization at the cytosol and some membrane structures (Fig 2A, upper right panels). To identify the membrane to which the GDP-bound form of Ypt7p is targeted, we followed several membrane markers and observed co-localization with Sec63p, a marker of the endoplasmic reticulum (ER) (Fig 2A, lower panels). This finding is consistent with the observation that Ypt7p is targeted to the ER membrane in the *mon1*Δ or *ccz1*Δ mutant [34]. Accordingly, we concluded that Ypt7p is localized at the vacuolar membrane in the active state whereas it is localized in the cytosol or on the ER membrane in the inactive state.

We next examined the active/inactive state of Ypt7p in the *vps21*Δ mutant. Unexpectedly, expression of GFP-Ypt7p in the *vps21*Δ mutant caused moderate vacuolar fragmentation and mis-localization of Ypt7p in the cytosol with a punctate structure, but additional expression of exogenous Ypt7p led to recovery of GFP-Ypt7p localization and the vacuolar morphology (Fig 2B). This suggests that N-terminal GFP tagging partially perturbs the function of Ypt7p. Importantly, vacuolar localization of GFP-Ypt7p in the *vps21*Δ mutant suggests that Ypt7p is activated in the absence of Vps21p. To further examine the localization of Ypt7p in mutant cells, we expressed GFP-Ypt7p with an exogenous single-copy *YPT7* gene in cells lacking a subunit of the CORVET, HOPS or SNARE complex. Interestingly, we found that GFP-Ypt7p showed partial relocation to the cytosol in the *vps3*Δ and *pep12*Δ mutants, but did not show cytosolic localization in the *vps41*Δ and *vam3*Δ mutants (Fig 2C and 2D). In the *vps33*Δ and *pep12*Δ *vam3*Δ mutants, GFP-Ypt7p also did not show cytosolic localization, but exhibited a punctate localization in the region of FM4-64 accumulation (Fig 2C). These observations indicate that Ypt7p is fully or partially activated in these mutant cells. In *mon1*Δ mutant, Ypt7p was localized at the vacuole but partially relocalized to the cytosol (Fig 2C and 2D). Taken together with the observation that Mon1p is slightly localized at the prevacuolar compartment in *rab5*Δ cells (Fig 1D), this result suggests that the Mon1-Ccz1 GEF complex partially contributes to the nucleotide exchange of Ypt7p in the absence of yeast Rab5s. Since one of the key effectors of Rab5 on early endosomes is the type III PI(3)-kinase, Vps34 [35], which produces phosphatidylinositol-3-phosphate (PtdIns(3)P) that is required for recruitment of the Mon1-Ccz1 complex and Rab7 [36], we examined intracellular PtdIns(3)P levels in *vps21*Δ and *mon1*Δ mutants. We detected the localization of PtdIns(3)P by FYVE-GFP [37], and found that PtdIns(3)P is substantially produced in these mutants (Fig 2E). Similar to GFP-Ypt7p localization, Vps21p also showed partial relocation to the cytosol in the *vps3*Δ mutant, but did not show cytosolic localization in other mutants (Fig 2F). We note that GFP-Vps21p is partially localized on the vacuolar membrane in the *ypt7*Δ mutant, as shown previously (Fig 2G) [38].

### *vps21*Δ *ypt7*Δ double mutant shows defective convergence of the CPY pathway with the AP-3 pathway

**Fig 3:**
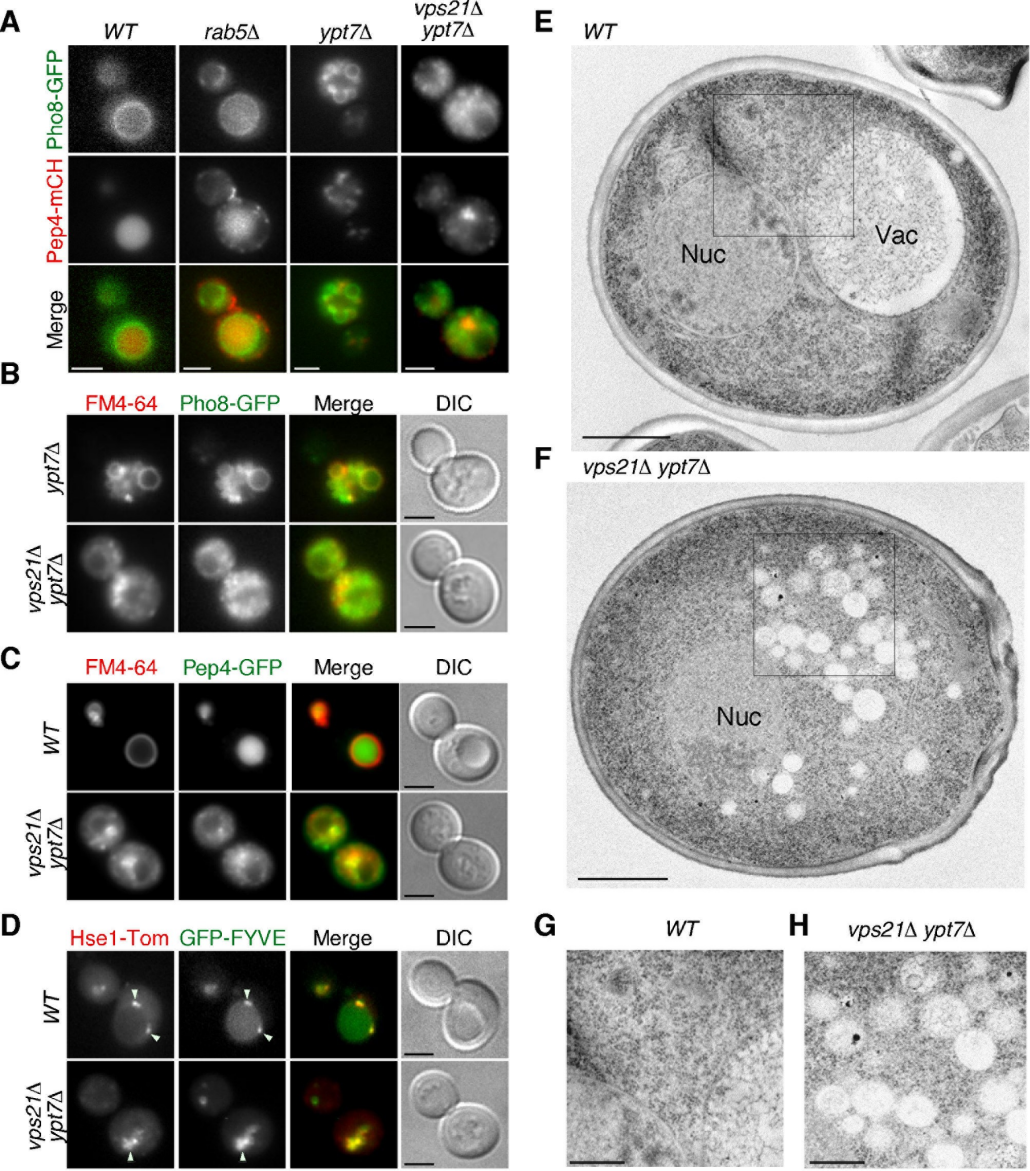
Convergence of the CPY pathway with the AP-3 pathway in *vps21*Δ *ypt7*Δ cells. (A) Localization of Pho8-GFP and Pep4-mCherry in *vps21*Δ and *ypt7*Δ cells. Cells expressing Pho8-GFP and Pep4-mCherry were grown to early to mid-logarithmic phase in YPD medium at 25°C and observed by fluorescence microscopy. Merged images of GFP and mCherry channels are shown in the lower panel. (B) Localization of Pho8-GFP in wild-type and *vps21*Δ *ypt7*Δ cells. Cells were labeled with FM4-64 and observed as described above. (C) Convergence of the endocytic pathway with the CPY pathway in wild-type and *vps21*Δ *ypt7*Δ cells. The images were acquired at 4h after labeling with 200 μM FM4-64. (D) Localization of Hse1-tdTomato and GFP-fused FYVE domain (EEA1) in wild-type and *vps21*Δ *ypt7*Δ cells. (E, F) Ultrastructure of vacuole(s) observed in wild-type and *vps21*Δ *ypt7*Δ cells. Cells were grown at 25°C, fixed using propane jet freezing method and processed for electron microscopic analysis. (G, H) Higher magnification views of the boxed areas in wild-type (E) and *vps21*Δ *ypt7*Δ (F) cells. Scale bars: 2.5 μm (A-D), 1 μm (E, F), 0.5 μm (G, H).

The finding that the *vps21*Δ *ypt7*Δ double mutant exhibits a much more abnormal vacuolar morphology than the *vps21*Δ or *ypt7*Δ single mutant suggested that function of these Rabs might be required for vesicle trafficking pathway(s) other than the CPY pathway. To investigate this, we examined the effect of deleting the *VPS21* and *YPT7* genes on the VPS and AP-3 pathways, using mCherry-tagged Pep4p, a vacuolar protease, as a marker of the CPY pathway, and GFP-tagged Pho8p, a vacuolar alkaline phosphatase, as a marker of the AP-3 pathway [39, 40]. In wild-type cells, these markers localized at the vacuole or vacuolar membrane (Fig 3A). In agreement with previous observations that the Rab5-disrupted cell exhibits a severe defect in the CPY pathway but shows little abnormality in the AP-3 pathway [21], Pep4-mCherry was ectopically localized to multiple punctate compartments in the cytosol, whereas Pho8-GFP was transported normally to the vacuolar membrane in the *rab5*Δ mutant (Fig 3A). In the *ypt7*Δ mutant, Pep4-mCherry and Pho8-GFP were likely to be transported to fragmented vacuoles and they partially colocalized (Fig 3A). To further examine effect of *YPT7* gene deletion on the AP-3 pathway, we compared localization of Pho8-GFP with FM4-64 staining. As expected, we observed that Pho8-GFP mostly colocalizes with FM4-64-labeled fragmented vacuoles in the *ypt7*Δ mutant (Fig 3B and S1 Movie), indicating that transport of Pho8-GFP to the vacuole via the AP-3 pathway is not impaired in the *ypt7*Δ mutant.

Interestingly, we found that in the *vps21*Δ *ypt7*Δ double mutant, Pep4-mCherry and Pho8-GFP exhibited clearly distinct localization: Pep4-mCherry showed punctate localization, which is clearly distinct from the class E compartment that is an aberrant prevacuolar endocytic compartment [22], beside the nucleus whereas Pho8-GFP showed small particulate localization in the cytosol (Fig 3A) and rarely colocalizes with FM4-64-labeled puncta (Fig 3B and S1 Movie). These results suggest that both of the CPY and AP-3 pathway might be impaired in the *vps21*Δ *ypt7*Δ mutant. In contrast, FM4-64 and Pep4-GFP accumulated in the same region beside the nucleus (Fig 3C). We also found that tdTomato-tagged Hse1p, a marker of the early-to-late endosome [21], and GFP-FYVE, a marker of the PtdIns(3)P residing late endosome and vacuole [37], accumulated in a similar region (Fig 3D).

Next, using electron microscopy, we explored the ultrastructure of the region where FM4-64 and Pep4-GFP accumulated in the *vps21*Δ *ypt7*Δ mutant. Numerous small vesicle structures were observed beside the nucleus in the *vps21*Δ *ypt7*Δ mutant (Fig 3F–3H), whereas in wild-type cells such structures were rarely detected (Fig 3E–3G). The vesicles accumulating beside the nucleus in the mutant were smaller than the fragmented vacuoles observed in mutants lacking the HOPS or SNARE subunit (Fig 1H–1J). On the basis of observations using FM4-64 and other markers, these structures appear to be vesicles derived from the endocytic and CPY pathways.

### Defective transport of AP-3-coated vesicles in *vps21*Δ *ypt7*Δ double mutant

**Fig 4:**
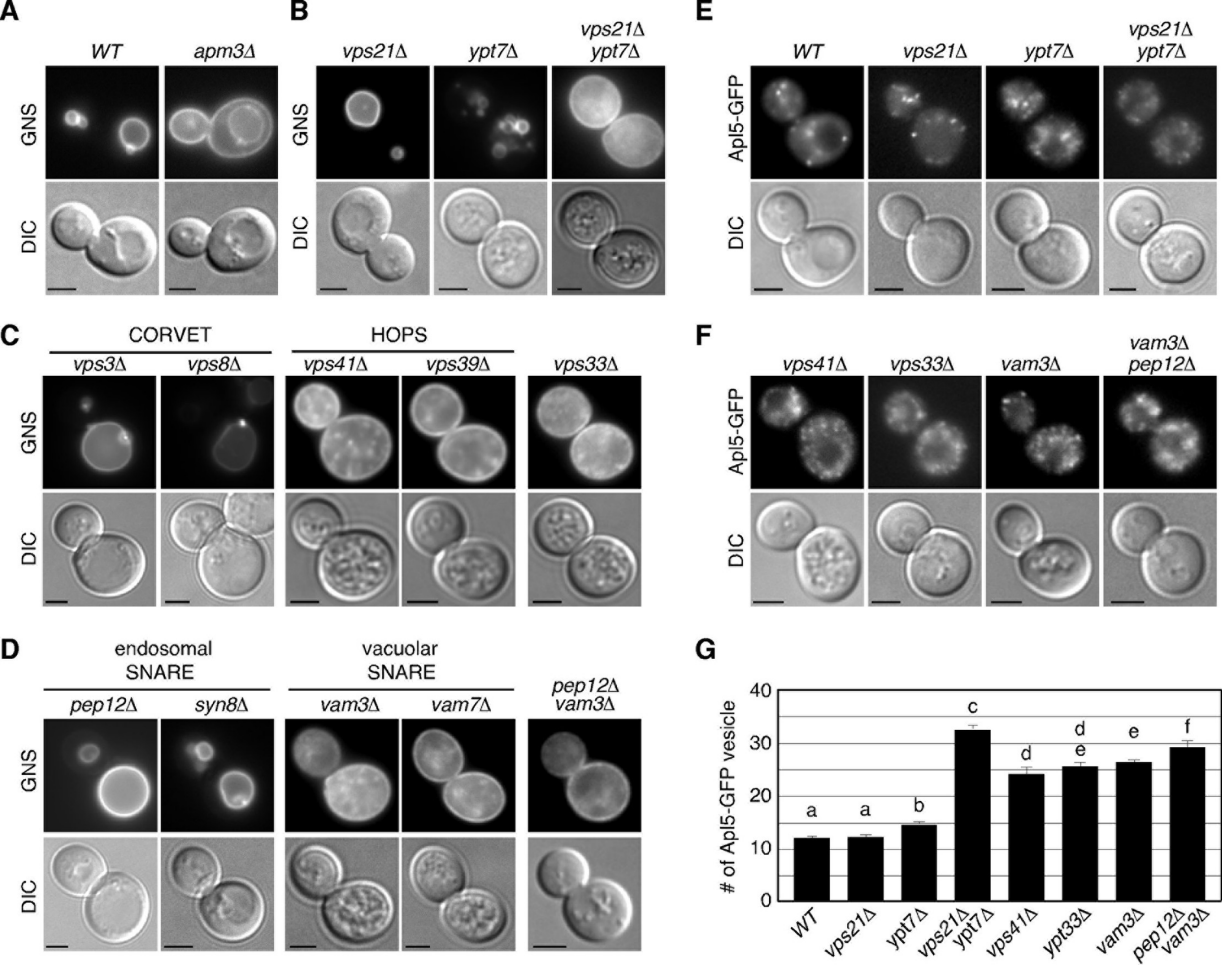
Defect of the AP-3 pathway in *vps21*Δ *ypt7*Δ mutant. (A-D) Analysis of the AP-3 pathway in wild-type and mutant cells. Cells expressing GFP–Nyv1–Snc1-TMD (GNS) fusion protein were grown to early to mid-logarithmic phase at 25°C and observed by fluorescence microscopy and DIC. (E, F) Localization of Apl5-GFP in wild-type and mutant cells. (G) Quantification of the number of Apl5-GFP positive vesicles in indicated cells displayed in (E) and (F). Data show the mean ± SEM of three experiments, with 50 cells per experiment. Different letters indicate significant difference at *p* < 0.05 (S2 Table) (One-way ANOVA with Tukey’s post-hoc test). Scale bars, 2.5 μm.

We next utilized the chimeric protein GFP–Nyv1–Snc1-TMD (GNS), which accumulates at the plasma membrane if the AP-3 pathway is defective (Fig 4A) [41]. Consistent with the result obtained using Pho8-GFP as a marker (Fig 3A), we found that the *vps21*Δ *ypt7*Δ double mutant showed accumulation of GNS at the plasma membrane (Fig 4B). Interestingly, the *vps21*Δ *ypt7*Δ mutant showed a severe defect in trafficking of the AP-3 pathway, even though the pathway is almost intact in each single mutant (Fig 4B). Localization of GNS in the *vps21*Δ *ypt7*Δ mutant was different from that in mutants lacking a subunit of the HOPS (*vps41*Δ or *vps39*Δ) or SNARE complex (*vam3*Δ or *vam7*Δ), in which GNS showed punctate localization in addition to localization at the plasma membrane (Fig 4C and 4D). In contrast, GNS localization in the *vps33*Δ or *vam3*Δ *pep12*Δ mutant was mostly observed at the plasma membrane, similar to that in the *vps21*Δ *ypt7*Δ mutant (Fig 4D).

We also utilized GFP-fused Apl5p, an AP-3 complex subunit localizing at the TGN and transport vesicles. Apl5-GFP was observed in the cytoplasm as multiple small puncta in wild-type cells (Fig 4E). The number of Apl5-GFP-labeled puncta was unchanged in *vps21*Δ or *ypt7*Δ mutant, but significantly increased in the *vps21*Δ *ypt7*Δ double mutant (Fig 4E–4G). Similar results were obtained in mutants lacking a subunit of the HOPS or SNARE complex (Fig 4F and 4G), suggesting that transport of the AP-3-coated vesicle to the vacuole is impaired in these mutant cells.

## Discussion

**Fig 5:**
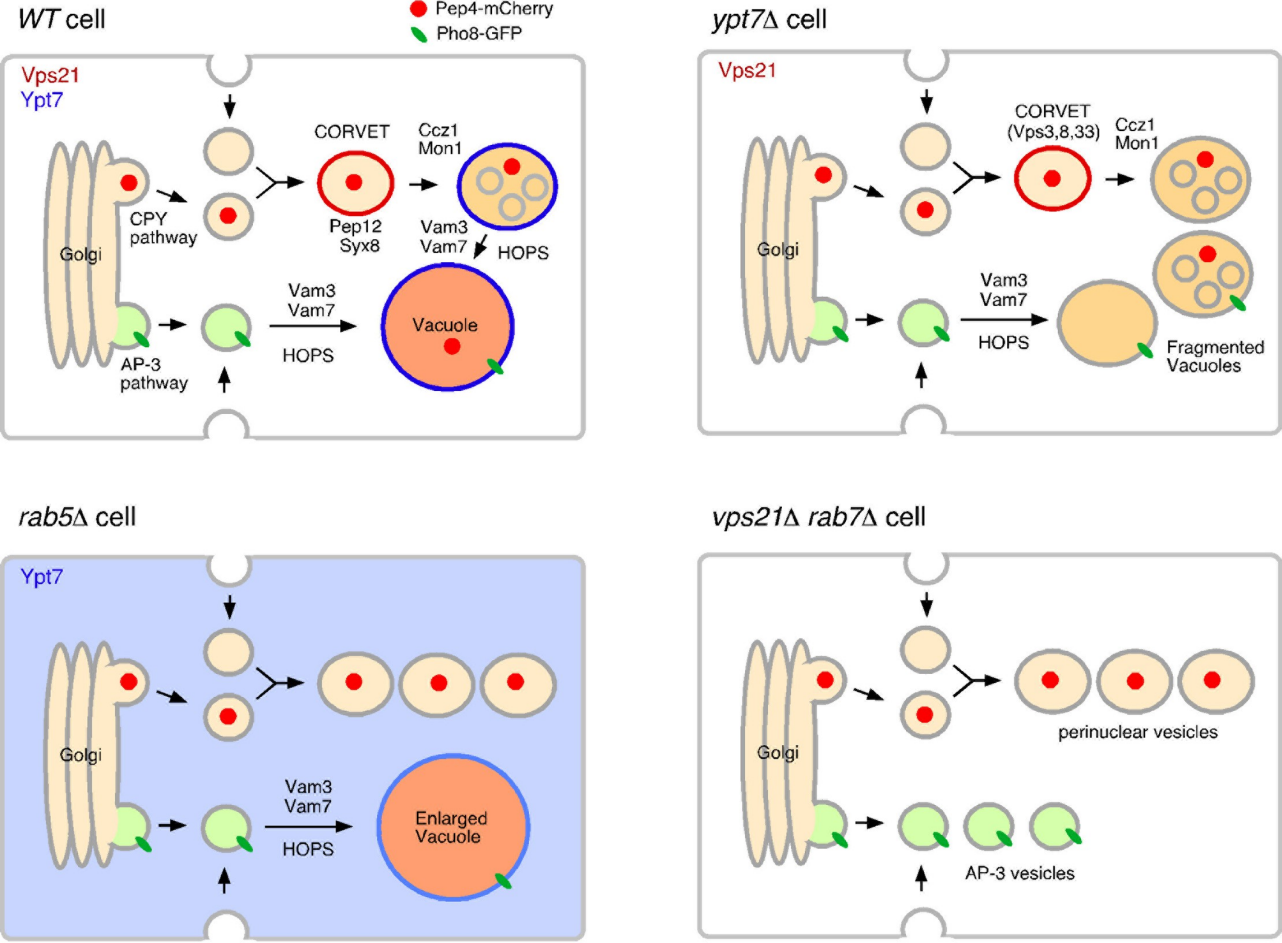
Model of vacuole delivery pathways in wild-type, *vps21*Δ and *ypt7*Δ cells. Convergence of the endocytic and CPY pathways occur at an early stage of endocytosis, independently of Rab5. *vps21*Δ *ypt7*Δ double mutant has defect in the trafficking of the AP-3 and CPY pathways although the AP3 pathway is mostly intact in each *vps21*Δ or *ypt7*Δ single mutant. See details in the text.

On the basis of the data presented above and in previous studies, we propose that activity of yeast Rab7, Ypt7p, is regulated in both the Rab5-dependent and -independent pathway. We previously reported that convergence of the endocytic and CPY pathways occurs before yeast Rab5s function, and that the endocytic pathway intersects separately with the CPY and AP-3 pathways (Fig 5) [21]. In Rab5-disrupted cells, therefore, transport intermediates derived from the endocytic and CPY pathways accumulated in the cytosol, whereas vesicles derived from the intact AP-3 pathway were able to fuse with the vacuole (Fig 5). In this step, Ypt7p is recruited to the vacuolar membrane to mediate the fusion. In the *ypt7*Δ mutant, convergence of the CPY and AP-3 pathways could partially occur, leading to formation of relatively large vesicles (Fig 5). The *vps21*Δ *ypt7*Δ double mutant exhibited accumulation of smaller vesicles beside the nucleus. Considering the phenotypic similarity of the *vps21*Δ *ypt7*Δ mutant to the class C *vps* mutant, the *vps21*Δ *ypt7*Δ mutant might have defects at multiple stages of vesicular trafficking, including convergence of the CPY and AP-3 pathways (Fig 5).

The *vps21*Δ *ypt7*Δ double mutant seems to possess a defect similar to that of the *vps33*Δ or *vam3*Δ *pep12*Δ double mutant. Vps33p is an essential core component of the CORVET and HOPS complexes, and is required for multiple stages of the endocytic and CPY pathways, including early-to-late endosome transition and fusion of the late endosome and vacuole [17, 25]. Additionally, mutants lacking the HOPS subunits exhibit a defect in the AP-3 pathway. Therefore, the *vps33*Δ mutant has defects in the endocytic, CPY and AP-3 pathways. The double SNARE mutant, *vam3*Δ *pep12*Δ, exhibits a vacuolar phenotype similar to that of the *vps33*Δ mutant [25]. Both the *vam3*Δ and *pep12*Δ single mutants have been shown to have a severe defect in the CPY pathway [24]. It has also been reported that Vam3p is required for the AP-3 complex-mediated transport of Pho8p [27]. These observations suggest that defects in all three of these pathways have a severe impact on vacuolar biogenesis.

We demonstrated that the *vps21*Δ *ypt7*Δ mutant has a severe defect in the AP-3 pathway, although the defect in the pathway exhibited by each single mutant alone is very slight. This suggests that Vps21p directly or indirectly functions in a redundant manner with Ypt7p in the AP-3 pathway. Ypt7p is reported to interact with Vps41p and mediate fusion of the AP-3 vesicles to the vacuole [42]. One possibility is that in the absence of Ypt7p, Vps21p might be able to mediate this fusion step instead of Ypt7p via, for example, the i-CORVET complex that contains Vps41p instead of Vps8p. Localization of Vps21p is regulated by a Rab-GAP, Gyp3p, and deletion of the *GYP3* gene changes the localization of Vps21p from the endosomal compartment to the vacuolar membrane [43, 44]. According to the Rab countercurrent model of GAP recruitment [45], Ypt7p could recruit Gyp3p to the vacuole and regulate Vps21p dissociation from the vacuole. In support of this idea, previous studies have demonstrated interaction between Ypt7p and Gyp3p [43]. Additionally, we and other group observed that Vps21p is partially localized on the vacuolar membrane in the *ypt7*Δ mutant, suggesting that Ypt7p is required for Vps21p inactivation [38]. Thus, Vps21p could function in a redundant manner with Ypt7p in the fusion step of the AP-3 vesicles to the vacuole.

We demonstrated that Ypt7p is able to localize on the vacuolar membrane in *vps21*Δ and *mon1*Δ mutants. Consistent with these observations, previous studies demonstrated that Ypt7p is targeted to the vacuolar membrane in the absence of the Mon1-Ccz1 complex [34]. These results suggest that the upstream Vps21p and GEF complex are not only determinants of Ypt7p and additional factors regulate Ypt7p targeting on the vacuolar membrane. One possible candidate might be Vps39p, which directly binds to the GDP-bound forms of Ypt7p [46]. Another possible candidate is PtdIns(3)P, which is produced by the type III PI(3)-kinase Vps34p on endosomal membrane and plays important role in the recruitment of Mon1-Ccz1 complex and subsequent Ypt7p targeting on the vacuolar membrane [47]. We demonstrated that PtdIns(3)P is substantially produced in *vps21*Δ and *mon1*Δ mutants. Many Rab5 and Rab7 effectors are known to interact with PtdIns(3)P, suggesting a possibility that Ypt7p might be targeted on the vacuole directly or indirectly via PtdIns(3)P.

## Supporting information

S1 Movie Localization of Pho8-GFP and FM4-64-labeleded compartments in wild-type and *vps21*Δ *ypt7*Δ cells. Cells expressing Pho8-GFP were grown to early to mid-logarithmic phase in YPD medium at 25°C, labeled with FM4-64, and imaged for 1 min. Interval between frames is 2 sec. For best viewing, movies should be played in the “loop” mode.
(MOV)

S1 Table Yeast strains used in this study. (DOCX)

S2 Table p values for all ANOVA tests. (XLSX)
