# Supplementary material for: Rab5-independent activation and function of yeast Rab7-like protein, Ypt7p, in the AP-3 pathway
Source: PLoS One. 2019 Jan 25;14(1):e0210223. doi: 10.1371/journal.pone.0210223 (PMC6347229; doi:10.1371/journal.pone.0210223)
Supplement: S1 Table — (DOCX) [file pone.0210223.s002.docx]

**S1 Table.** Yeast Strains used in this study

Strain Genotype Source

BY4741 *Mat***a** *his3*Δ*1* *leu2*Δ*0* *lys2*Δ*0 ura3*Δ*0 met15*Δ*0* GE Healthcare Dharmacon

BY4741 *vps21* *Mat***a** *his3*Δ*1* *leu2*Δ*0* *lys2*Δ*0 ura3*Δ*0* *met15*Δ*0 vps21*Δ::*KanMX* GE Healthcare Dharmacon

BY4741 *ypt7* *Mat***a** *his3*Δ*1* *leu2*Δ*0* *lys2*Δ*0 ura3*Δ*0* *met15*Δ*0 ypt7*Δ::*KanMX* GE Healthcare Dharmacon

BY4741 *mon1* *Mat***a** *his3*Δ*1* *leu2*Δ*0* *lys2*Δ*0 ura3*Δ*0* *met15*Δ*0 mon1*Δ::*KanMX* GE Healthcare Dharmacon

BY4741 *vps3* *Mat***a** *his3*Δ*1* *leu2*Δ*0* *lys2*Δ*0 ura3*Δ*0* *met15*Δ*0 vps3*Δ::*KanMX* GE Healthcare Dharmacon

BY4741 *vps8* *Mat***a** *his3*Δ*1* *leu2*Δ*0* *lys2*Δ*0 ura3*Δ*0* *met15*Δ*0 vps8*Δ::*KanMX* GE Healthcare Dharmacon

BY4741 *vps41* *Mat***a** *his3*Δ*1* *leu2*Δ*0* *lys2*Δ*0 ura3*Δ*0* *met15*Δ*0 vps41*Δ::*KanMX* GE Healthcare Dharmacon

BY4741 *vps33* *Mat***a** *his3*Δ*1* *leu2*Δ*0* *lys2*Δ*0 ura3*Δ*0* *met15*Δ*0 vps33*Δ::*KanMX* GE Healthcare Dharmacon

BY4741 *syn8* *Mat***a** *his3*Δ*1* *leu2*Δ*0* *lys2*Δ*0 ura3*Δ*0* *met15*Δ*0 syn8*Δ::*KanMX* GE Healthcare Dharmacon

BY4741 *vam3* *Mat***a** *his3*Δ*1* *leu2*Δ*0* *lys2*Δ*0 ura3*Δ*0* *met15*Δ*0 vam3*Δ::*KanMX* GE Healthcare Dharmacon

BY4741 *vam7* *Mat***a** *his3*Δ*1* *leu2*Δ*0* *lys2*Δ*0 ura3*Δ*0* *met15*Δ*0 vam7*Δ::*KanMX* GE Healthcare Dharmacon

JJTY1413 *Mat*α *his3-*Δ*200 leu2-3, 112 ura3-52 lys2-801 GFP-YPT7(Q68L)*::*HIS3* This study

JJTY1428 *Mat***a** *his3-Δ200 leu2-3, 112 ura3-52 lys2-801 GFP-YPT7(T22N)*::*HIS3 bar1Δ*::*LEU2* This study

JJTY1631 *Mat*α *his3*-Δ*200* *leu2-3*, *112* *ura3-52 lys2-801,GFP-Pho8*::*HIS3 Pep4-mCherry*::*URA* This study

JJTY1634 *Mat***a** *his3-Δ200 leu2-3, 112 ura3-52 lys2-801 bar1Δ*::*cgLEU2 ypt7Δ*::*KanMX GFP-YPT51*::*HIS3* This study

JJTY1971 *Mat***a** *his3*-Δ*200* *leu2-3*, *112* *ura3-52* *lys2-801* *vps21*Δ::*KanMX GFP-YPT7*::*HIS3 bar1*Δ::*LEU2* This study

JJTY2010 *Mat***a** *his3*Δ*1* *leu2*Δ*0* *lys2*Δ*0 ura3*Δ*0 bar1*Δ::*LEU2 PEP4-GFP*:: *HIS3* This study

JJTY2049 *Mat***a** *his3*Δ*1* *leu2*Δ*0* *lys2*Δ*0 ura3*Δ*0* *vps21*Δ::*KanMX ypt52*Δ::*KanMX ypt53*Δ::*KanMX* This study

JJTY2444 *Mat*α *his3*Δ*1* *leu2*Δ*0* *lys2*Δ*0 ura3*Δ*0 vps21*Δ::*KanMX ypt7*Δ::*KanMX* This study

JJTY2716 *Mat***a** *his3*Δ*1* *leu2*Δ*0* *lys2*Δ*0 ura3*Δ*0 vps21*Δ*ypt52*Δ*ypt53*Δ::*KanMX bar1*Δ::*cgLEU2*

*GFP-Pho8*::*HIS3 pep4-mCherry*::*URA3* This study

JJTY2466 *Mat*α *his3*Δ*1* *leu2*Δ*0* *lys2*Δ*0 ura3*Δ*0 ypt7*Δ::*KanMX GFP-PHO8*::*HIS3 PEP4-mCherry*::*URA3* This study

JJTY3059 *Mat***a** *his3*Δ*1* *leu2*Δ*0* *lys2*Δ*0 ura3*Δ*0 bar1*Δ::*LEU2* [*pRS416-GFP-GNS*] This study

JJTY3060 *Mat***a** *his3*Δ*1* *leu2*Δ*0* *lys2*Δ*0 ura3*Δ*0 bar1*Δ::*LEU2 vps21*Δ::*KanMX* [*pRS416-GFP-GNS*] This study

JJTY3103 *Mat***a** *his3*-Δ*200* *leu2-3*, *112* *ura3-52* *bar1*Δ::*LEU2 GFP-Vps41*::*HIS3* This study

JJTY3112 *Mat***a** *his3*Δ*1* *leu2*Δ*0* *lys2*Δ*0 ura3*Δ*0 met15*Δ*0 vps41*Δ::*KanMX* [*pRS416-GFP-GNS*] This study

JJTY3341 *Mat***a** *his3*Δ*1* *leu2*Δ*0* *lys2*Δ*0 ura3*Δ*0 met15*Δ*0 apm3*Δ::*KanMX* [*pRS416-GFP-GNS*] This study

JJTY3366 *Mat***a** *his3*Δ*1* *leu2*Δ*0* *lys2*Δ*0 ura3*Δ*0* *vps21*Δ::*KanMX ypt52*Δ::*KanMX ypt53*Δ::*KanMX*

*GFP-VPS41*::*HIS3* This study

JJTY3638 *Mat***a** *his3*Δ*1* *leu2*Δ*0* *ura3*Δ*0* *lys2*Δ*0 GFP-Vps21*::*HIS3* This study

JJTY4499 *Mat***a** *his3Δ1 leu2Δ0 ura3Δ0 lys2Δ0 met15*Δ*0 vps3Δ*::*Kan GFP-VPS21*::*HIS3* This study

JJTY4500 *Mat***α** *his3Δ1 leu2Δ0 ura3Δ0 vps8Δ*::*KanMX GFP-VPS51*::*HIS3* This study

JJTY4502 *Mat***a** *his3Δ1 leu2Δ0 ura3Δ0 lys2Δ0 vps33Δ*::*KanMX GFP-VPS21*::*HIS3* This study

JJTY4503 *Mat***a** *his3Δ1 leu2Δ0 ura3Δ0 lys2Δ0 vps41Δ*::*KanMX GFP-VPS21*::*HIS3* This study

JJTY5070 *Mat***a** *his3*-Δ*200* *leu2-3*, *112* *ura3-52* *lys2-8010 vps39Δ*::*HphMX4* This study

JJTY6253 *Mat***a** *his3*Δ*1* *leu2*Δ*0* *lys2*Δ*0 ura3*Δ*0 GFP-YPT7*::*HIS3* This study

JJTY6271 *Mat***a** *his3*Δ*1* *leu2*Δ*0* *lys2*Δ*0 ura3*Δ*0 GFP-MON1*::*HIS3* This study

JJTY6273 *Mat***a** *his3*Δ*1* *leu2*Δ*0* *lys2*Δ*0 ura3*Δ*0* *vps21*Δ::*KanMX ypt52*Δ::*KanMX ypt53*Δ::*KanMX*

*GFP-MON1*::*HIS3* This study

JJTY7413 *Mat***a** *his3*Δ*1* *leu2*Δ*0* *lys2*Δ*0 ura3*Δ*0 vps21*Δ::*KanMX ypt7*Δ::*KanMX* [*pRS416-GFP-GNS*] This study

JJTY7429 *Mat***a** *his3*Δ*1* *leu2*Δ*0* *lys2*Δ*0 ura3*Δ*0* *vps21*Δ::*KanMX ypt52*Δ::*KanMX ypt53*Δ::*KanMX*

*bar1*Δ::*LEU2 GFP-YPT7*::*HIS3* [*pRS316-YPT7*] This study

JJTY7451 *Mat***a** *his3*Δ*1* *leu2*Δ*0* *lys2*Δ*0 ura3*Δ*0 vps41*Δ::*KanMX GFP-YPT7*::*HIS* [*pRS316-YPT7*] This study

JJTY7459 *Mat***a** *his3*Δ*1* *leu2*Δ*0* *lys2*Δ*0 ura3*Δ*0 vam3*Δ::*KanMX* [*pRS416-GFP-GNS*] This study

JJTY7460 *Mat***a** *his3*Δ*1* *leu2*Δ*0* *lys2*Δ*0 ura3*Δ*0 vam7*Δ::*KanMX* [*pRS416-GFP-GNS*] This study

JJTY7464 *Mat***a** *his3*Δ*1* *leu2*Δ*0* *lys2*Δ*0 ura3*Δ*0 vps39*Δ::*HphMX* [*pRS416-GFP-GNS*] This study

JJTY7465 *Mat***a** *his3*Δ*1* *leu2*Δ*0* *lys2*Δ*0 ura3*Δ*0 vp33*Δ::*KanMX* [*pRS316-YPT7*] This study

JJTY7820 *Mat***a** *his3*Δ*1* *leu2*Δ*0* *lys2*Δ*0 ura3*Δ*0 vps21*Δ::*KanMX ypt7*Δ::*KanMX Halo-PHO8*::*URA3*

*APL5-GFP*::*HIS3* This study

JJTY7825 *Mat***a** *his3*Δ*1* *leu2*Δ*0* *lys2*Δ*0 ura3*Δ*0 vps21*Δ::*KanMX ypt7*Δ::*KanMX HSE1-tdTomato*::*URA3*

*[pRS303-GFP-FYVE]* This study

JJTY7862 *Mat***a** *his3*Δ*1* *leu2*Δ*0* *lys2*Δ*0 ura3*Δ*0 ypt7*Δ::*KanMX* [*pRS416-GFP-GNS*] This study

JJTY7879 *Mat***a** *his3*Δ*1* *leu2*Δ*0* *lys2*Δ*0 ura3*Δ*0 Apl5-GFP*::*HIS3* This study

JJTY7901 *Mat***a** *his3-Δ200 leu2-3, 112 ura3-52 lys2-801 bar1Δ*::*LEU2 1GFP-YPT7(T22N)*::*HIS3*

*SEC63-mCherry*::*URA3* This study

JJTY8175 *Mat***a** *his3*Δ*1* *leu2*Δ*0* *lys2*Δ*0 ura3*Δ*0 ypt7*Δ::*KanMX vps21*Δ::*KanMX GFP-Pho8*::*LEU2*

*Pep4-mCherry*::*URA3* This study

JJTY8181 *Mat***a** *his3*Δ*1* *leu2*Δ*0* *lys2*Δ*0 ura3*Δ*0 vps41*Δ::*KanMX Apl5-GFP*::*HIS3* This study

JJTY8185 *Mat***a** *his3*Δ*1* *leu2*Δ*0* *lys2*Δ*0 ura3*Δ*0 vps33*Δ::*KanMX Apl5-GFP*::*HIS3* This study

JJTY8187 *Mat***a** *his3*Δ*1* *leu2*Δ*0* *lys2*Δ*0 ura3*Δ*0 vps33*Δ::*KanMX GFP-YPT7*::*HIS3* [*pRS316-YPT7*] This study

JJTY8198 *Mat***a** *his3*Δ*1* *leu2*Δ*0* *lys2*Δ*0 ura3*Δ*0 mon1*Δ::*KanMX GFP-YPT7*::*HIS3* [*pRS316-YPT7*] This study

JJTY8203 *Mat***a** *his3*Δ*1* *leu2*Δ*0* *lys2*Δ*0 ura3*Δ*0 GFP-YPT7*::*HIS3* [*pRS316-YPT7*] This study

JJTY8209 *Mat***a** *his3*Δ*1* *leu2*Δ*0* *lys2*Δ*0 ura3*Δ*0 vps21*Δ::*KanMX Apl5-GFP*::*HIS3* This study

JJTY8212 *Mat***a** *his3*Δ*1* *leu2*Δ*0* *lys2*Δ*0 ura3*Δ*0 vps21*Δ::*KanMX mon1*Δ::*LEU2* This study

JJTY8213 *Mat***a** *his3*Δ*1* *leu2*Δ*0* *lys2*Δ*0 ura3*Δ*0 vps21*Δ::*KanMX GFP-YPT7*::*HIS3* [*pRS316-YPT7*] This study

JJTY8219 *Mat***a** *his3*Δ*1* *leu2*Δ*0* *lys2*Δ*0 ura3*Δ*0 ypt7*Δ::*KanMX Apl5-GFP*::*HIS3* This study

JJTY8223 *Mat***a** *his3*Δ*1* *leu2*Δ*0* *lys2*Δ*0 ura3*Δ*0 vps3*Δ::*KanMX GFP-YPT7*::*HIS3* [*pRS316-YPT7*] This study

JJTY8224 *Mat***a** *his3*Δ*1* *leu2*Δ*0* *lys2*Δ*0 ura3*Δ*0 vps3*Δ::*KanMX* [*pRS416-GFP-GNS*] This study

JJTY8225 *Mat***a** *his3*Δ*1* *leu2*Δ*0* *lys2*Δ*0 ura3*Δ*0 vps8*Δ::*KanMX* [*pRS416-GFP-GNS*] This study

JJTY8226 *Mat***a** *his3*Δ*1* *leu2*Δ*0* *lys2*Δ*0 ura3*Δ*0 syn8*Δ::*KanMX* [*pRS416-GFP-GNS*] This study

JJTY8228 *Mat***a** *his3*Δ*1* *leu2*Δ*0* *lys2*Δ*0 ura3*Δ*0 vam3*Δ::*KanMX GFP-YPT7*::*HIS3* [*pRS316-YPT7*] This study

JJTY8271 *Mat***a** *his3*Δ*1* *leu2*Δ*0* *lys2*Δ*0 ura3*Δ*0* *lys2*Δ*0 pep12*Δ::*LEU2* This study

JJTY8272 *Mat***a** *his3*Δ*1* *leu2*Δ*0* *lys2*Δ*0 ura3*Δ*0 vam3*Δ::*KanMX pep12*Δ::*LEU2* This study

JJTY8273 *Mat***a** *his3*Δ*1* *leu2*Δ*0* *lys2*Δ*0 ura3*Δ*0 pep12*Δ::*LEU2 GFP-YPT7*::*HIS3* [*pRS316-YPT7*] This study

JJTY8274 *Mat***a** *his3*Δ*1* *leu2*Δ*0* *lys2*Δ*0 ura3*Δ*0 vam3*Δ::*KanMX pep12*Δ::*LEU2 GFP-YPT7*::*HIS3*

[*pRS316-YPT7*] This study

JJTY8275 *Mat***a** *his3*-Δ*200* *leu2-3*, *112* *ura3-52* *lys2-801 Hse1-tdTomato*::*URA3* This study

JJTY8276 *Mat*α *his3*Δ*1* *leu2*Δ*0* *lys2*Δ*0 ura3*Δ*0 ypt7*Δ::*KanMX vps21*Δ::*KanMX Pep4-GFP*::*HIS3* This study

JJTY8277 *Mat***a** *his3*Δ*1* *leu2*Δ*0* *lys2*Δ*0 ura3*Δ*0 pep12*Δ::*LEU2* [*pRS416-GFP-GNS*] This study

JJTY8278 *Mat***a** *his3*Δ*1* *leu2*Δ*0* *lys2*Δ*0 ura3*Δ*0 vam3*Δ::*KanMX APL5-GFP::HIS3* This study

JJTY8279 *Mat***a** *his3*Δ*1* *leu2*Δ*0* *lys2*Δ*0 ura3*Δ*0 vam3*Δ::*KanMX pep12*Δ::*LEU2 APL5-GFP::HIS3* This study

JJTY8281 *Mat***a** *his3*Δ*1* *leu2*Δ*0* *lys2*Δ*0 ura3*Δ*0* *met15*Δ*0 vam3*Δ::*KanMX pep12*Δ*::LEU2* [*pRS416-GFP-GNS*] This study
